# Effects of larval exposure to sublethal doses of ivermectin on adult fitness and susceptibility to ivermectin in *Anopheles gambiae* s.s.

**DOI:** 10.1186/s13071-023-05888-w

**Published:** 2023-08-21

**Authors:** Caroline Kiuru, Kelly Ominde, Martha Muturi, Lawrence Babu, Caroline Wanjiku, Carlos Chaccour, Marta Ferreira Maia

**Affiliations:** 1 Barcelona Institute for Global Health (ISGlobal), Hospital Clínic - Universitat de Barcelona, Rosello 132, 5ª 2ª, 08036 Barcelona, Spain; 2 Centro de Investigação em Saúde de Manhiça (CISM), Maputo, Mozambique; 3 Department of Biosciences, KEMRI Wellcome Trust Research Programme (KWTRP), Kilifi, 230-80108 Kenya; 4 Pwani University, Department of Biological Sciences and Pwani University Bioscience Research Centre (PUBReC), Kilifi, Kenya; 5 Facultad de Medicina, Universidad de Navarra, 31008 Pamplona, Spain; 6 Centro de Investigación Biomédica en Red de Enfermedades Infecciosas, Madrid, Spain; 7 Nuffield Department of Medicine, Centre for Tropical Medicine and Global Health, University of Oxford, Oxford, UK

**Keywords:** Ivermectin, Mosquito larvae, Adult fitness, *Anopheles gambiae* s.s.

## Abstract

**Background:**

The effects of ivermectin (endectocide) on mosquito survival make it a potential new malaria vector control tool. The drug can be administered to mosquito disease vectors through blood hosts that include humans and livestock. Its increased use may cause contamination of larval habitats, either directly through livestock excreta or indirectly through leaching or run-off from contaminated soil, albeit in sublethal doses. However, the effects of such exposure on immature stages and the subsequent adults that emerge are poorly understood. This study was undertaken to evaluate the impact of ivermectin exposure on *Anopheles gambiae* s.s. larvae and its effects on fitness and susceptibility to ivermectin in the emerging adults.

**Methods:**

Laboratory-reared *An. gambiae* s.s. (Kilifi strain) larvae were exposed to five different ivermectin concentrations; 0, 0.00001, 0.0001, 0.001, and 0.01 ppm, and larval survival was monitored to determine the appropriate sub-lethal dose. Concentrations with survival > 50% (0.00001 and 0.0001 ppm) were selected and used as the sub-lethal doses. The fecundity, fertility, and susceptibility to ivermectin of adults emerging after larval exposure to the sub-lethal doses were examined.

**Results:**

Overall, exposure of *An. gambiae* s.s. aquatic stages to ivermectin caused a dose-dependent reduction in larval survival irrespective of the stage at which the larvae were exposed. Exposure to ivermectin in the larval stage did not have an effect on either the number of eggs laid or the hatch rate. However, exposure of first/second-instar larvae to 0.0001 ppm and third/fourth-instar larvae to 0.001 ppm of ivermectin reduced the time taken to oviposition. Additionally, exposure to ivermectin in the larval stage did not affect susceptibility of the emerging adults to the drug.

**Conclusions:**

This study shows that contamination of larval habitats with ivermectin affects An. *gambiae* s.s. larval survival and could potentially have an impact on public health. However, there are no carry-over effects on the fecundity, fertility, and susceptibility of the emerging adults to ivermectin. In addition, this study shows that environmental exposure to ivermectin in the larval habitats is unlikely to compromise the efficacy of ivermectin in the emerging adults.

**Graphical Abstract:**

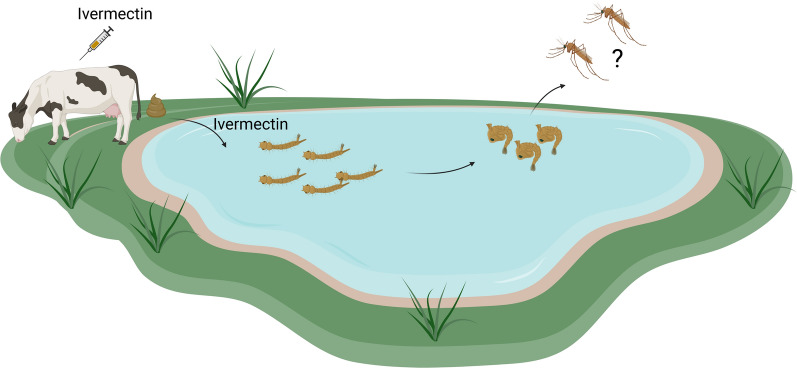

## Introduction

Since its discovery, ivermectin has been widely used as a parasiticide in both veterinary and human medicine [1]. The last 30 years have seen a scale-up in ivermectin coverage in sub-Saharan Africa through mass drug administration (MDA) campaigns for the control of onchocerciasis and lymphatic filariasis [2, 3]. In addition to its antiparasitic properties, ivermectin has been shown to reduce survival of adult female mosquitoes that blood-feed on an ivermectin-treated host [4, 5]. The ability to reduce survival of malaria vectors and in turn alter malaria transmission have led to ivermectin being considered a potential tool to combat malaria [6, 7]. A single mass drug administration (MDA) of ivermectin has been shown to reduce survival of *Anopheles gambiae* by 33% for a period of 1 week, sporozoite rates by 77% up to 2 weeks, and parity rates for > 2 weeks after drug administration [8]. Moreover, sub-lethal concentrations of the drug inhibit *Plasmodium* development in *An. gambiae*, further highlighting the usefulness of the drug in malaria transmission reduction [9]. The efficacy of ivermectin in reducing mosquito survival is dependent on the plasma concentration of ivermectin and the time that the lethal plasma concentration of ivermectin can be sustained [10]. To increase ivermectin efficacy, several pharmacological strategies have been proposed; (i) increasing ivermectin dosage, (ii) administering repeated doses, (iii) using long-lasting formulations of ivermectin, (iv) using pharmaco-enhancing to boost half-life in the mammal target or pharmacodynamics in the mosquito, and (v) Expanding ivermectin treated blood sources by targeting livestock [10–12]. While targeting livestock could increase the vector control coverage to zoophagic and exophagic vectors, it could potentially create a source for environmental contamination with ivermectin.

The fecal excreta of treated animals may contaminate both terrestrial and/or aquatic systems of the environment. Contamination of aquatic systems could expose mosquito larvae to ivermectin. However, the toxicity levels of ivermectin to immature *Anophelines* is not well documented [13]. While exposure to ivermectin in the adult stage is reported to reduce survival, fecundity, and fertility, we do not fully understand its effect in the larval stage [4, 5, 14, 15]. Considering that ivermectin could be used in livestock for malaria control, it is important to characterize how contamination of breeding sites may affect larval development and the fitness of the emerging adults [16, 17].

This study sought to evaluate the effect of the exposure of *An. gambiae* s.s. to sublethal doses of ivermectin during the larval stages as well as the fitness and susceptibility to ivermectin of the emerging adults.

## Methods

### Mosquitoes

Adult *An. gambiae* s.s. colonized at KEMRI-Wellcome Trust Research Programme insectary in 2011 from larvae collected in Mbogolo village of Kilifi county in Kenya were used for this study. This strain is fully susceptible to pyrethroids as it is routinely characterized quarterly for phenotypic resistance to pyrethroids using WHO tube tests. All experiments were carried out under insectary conditions at 28 ± 2 °C and 80 ± 10% relative humidity (RH) for rearing adults and at 30 ± 2 °C and 80 ± 10% RH for rearing larvae, under a 12 h light:12 h dark photoperiod. Adults were maintained on 10% glucose provided by soaking cotton wool pads in 10% glucose solution. Female adult mosquitoes, aged 2–5 days, were fed human blood through an artificial membrane to obtain eggs, which were the 86th filial generation since colonization. The eggs were collected on filter paper and immersed in deionized water for hatching. For first/second-instar larvae, hereafter referred to as L1/L2, sorting and counting of the larvae was done 2 days post-hatching, while for the third/fourth-instar larvae, hereafter referred to as L3/L4, sorting and counting was done 6 days post-hatching.

### Ivermectin stock solution

For all experiments, 1% injectable Ivermectin (Ivermet^®^) was used. It was diluted in deionized water to prepare 50 ml of a stock ivermectin solution of 1000 ng/ml. All stock solutions were freshly prepared during the experiments.

### Larval bioassay

About 100 larvae were counted and sorted into larval rearing trays and 250 ml deionized water containing the appropriate final concentration of ivermectin: 0.01, 0.001, 0.0001, or 0.00001 ppm was added. For controls, hereafter referred to as 0 ppm, only deionized water was added. This was done in triplicate for every concentration of ivermectin and replicated twice using two different batches of eggs yielding three technical replicates per biological replicate. In total, for every ivermectin treatment, ~ 600 L1/L2 and ~ 600 L3/L4 larvae were used. The larvae were maintained under normal rearing conditions at 30 ± 2 °C and 80 ± 10% relative humidity and maintained on equal weight of baby fish food (Tetramin^®^). At L1/L2 stages, larvae were fed 0.05 g fish food once daily, while at L3/L4 stages, larvae were fed 0.05 g fish food twice daily. Larval survival was monitored for 15 days, and pupae were collected daily for 3 days following the emergence of the first pupae. The daily number of pupae collected was counted, and the pupae were placed inside a 20 × 20 × 20-cm netted cage to eclose. To ensure that all the adults used in subsequent experiments were within the range of 3–5 days old, the pupation rate was censored at day 3 for all experimental conditions. Only pupae that emerged within the first 3 days were used in subsequent experiments. All pupae from the same larval rearing tray collected during the 3 day collection period were placed in the same cage. The emerging adult mosquitoes were maintained on 10% glucose solution. Blood feeding of the emerging adults was done 2 days after the last pupae were placed inside the cage; this ensured that the blood-fed adults were 3 to 5 days old. For all cages, blood feeding was done using 1 ml human blood in a membrane glass feeder.

### Oviposition and fecundity assay

Oviposition cups were prepared by layering the base of a 250 ml plastic cups with a cotton wool pad moistened with deionized water. The cotton wool pad was covered with a filter paper, and the cup opening/mouth was covered using an insecticide-free net fastened with a rubber band. Gently, gravid females were transferred to individual oviposition cups. The mosquitoes were maintained on a 10% glucose solution and oviposition monitored daily for 10 days. After confirming the presence of eggs, mosquitoes were removed and killed by placing them in a −20 °C freezer for about 5 min. The filter paper retrieved from the oviposition cup with eggs was moistened with deionized water, and the eggs were allowed to hatch. Using a two-place denominator counter, the eggs were counted under a stereo microscope; eggs were counted to obtain the number of eggs hatched on one denominator and non-hatched eggs on the other denominator. The hatched eggs were identified by their dislodged operculum [18]. The numbers of hatched and non-hatched eggs were recorded for each oviposition cup.

### Adult susceptibility bioassay

Female adult mosquitoes, aged 3–5 days old, emerging from larvae exposed to ivermectin and their control of larvae not exposed to ivermectin were placed in 1-l mosquito holding cups previously described in [19]. Mosquitoes from every larval exposure replicate were placed in a separate holding cup. The number of mosquitoes per holding cup ranged from 20 to 49. The mosquitoes were starved for 5 h before being membrane-fed on human blood spiked with ivermectin at a concentration of 11 ng/ml. For the non-exposed adult control, a similar amount of PBS was added to the blood in place of ivermectin. Mosquitoes were allowed to blood feed for about 45 min, after which, mosquitoes that were not fully engorged and dead mosquitoes were removed and recorded. As the amount of ivermectin taken by the mosquito is relative to the amount of blood consumed, only fully engorged mosquitoes were kept. This was done to standardize the amount of ivermectin consumed. Fully engorged mosquitoes were visually identified by a distended abdomen and presence of blood in the whole abdomen.

The adults were maintained at standard insectary conditions, 28 ± 2 °C and 80 ± 10% relative humidity. The adults were provided with 10% glucose and mortality monitored daily for 10 days. Dead mosquitoes were removed, counted, and recorded daily. For analysis, larvae not pre-exposed to ivermectin and not exposed to ivermectin as adults were used as the negative control. For the positive control, larvae not pre-exposed to ivermectin but exposed to ivermectin as adults were used.

### Data analysis

All data were entered in MS Excel. Kaplan-Meier survival analyses were performed using R software, version 4.1.0. (R Core Team 2020). Log-rank test using a 5% significance was used to compare overall survival. For pairwise comparisons, Bonferroni was used to correct for multiple comparisons. Generalized negative binomial model with a log link was fitted to determine the relationship between time (days) taken to oviposition and ivermectin concentration and biological replicates (Table 2). The relationship between oviposition and predictors (ivermectin concentration and replicates) was performed using multivariable logistic regression analysis (Table 3). The influence of ivermectin concentration on total eggs laid by individual adult mosquitoes was assessed using generalized linear regression (Table 4). Generalized linear logistic regression was used to fit the relationship between the proportion of eggs hatched as the outcome variable and independent factors (ivermectin concentration, replicate) (Table 5). Predictors with *p* < 0.05 were considered independently associated with the outcome variables. Statistical analyses were performed using Stata version 15.0 (Stata Corp., College Station, TX).

## Results

### Determination of sublethal concentration of ivermectin in *Anopheles* larvae

**Fig. 1:**
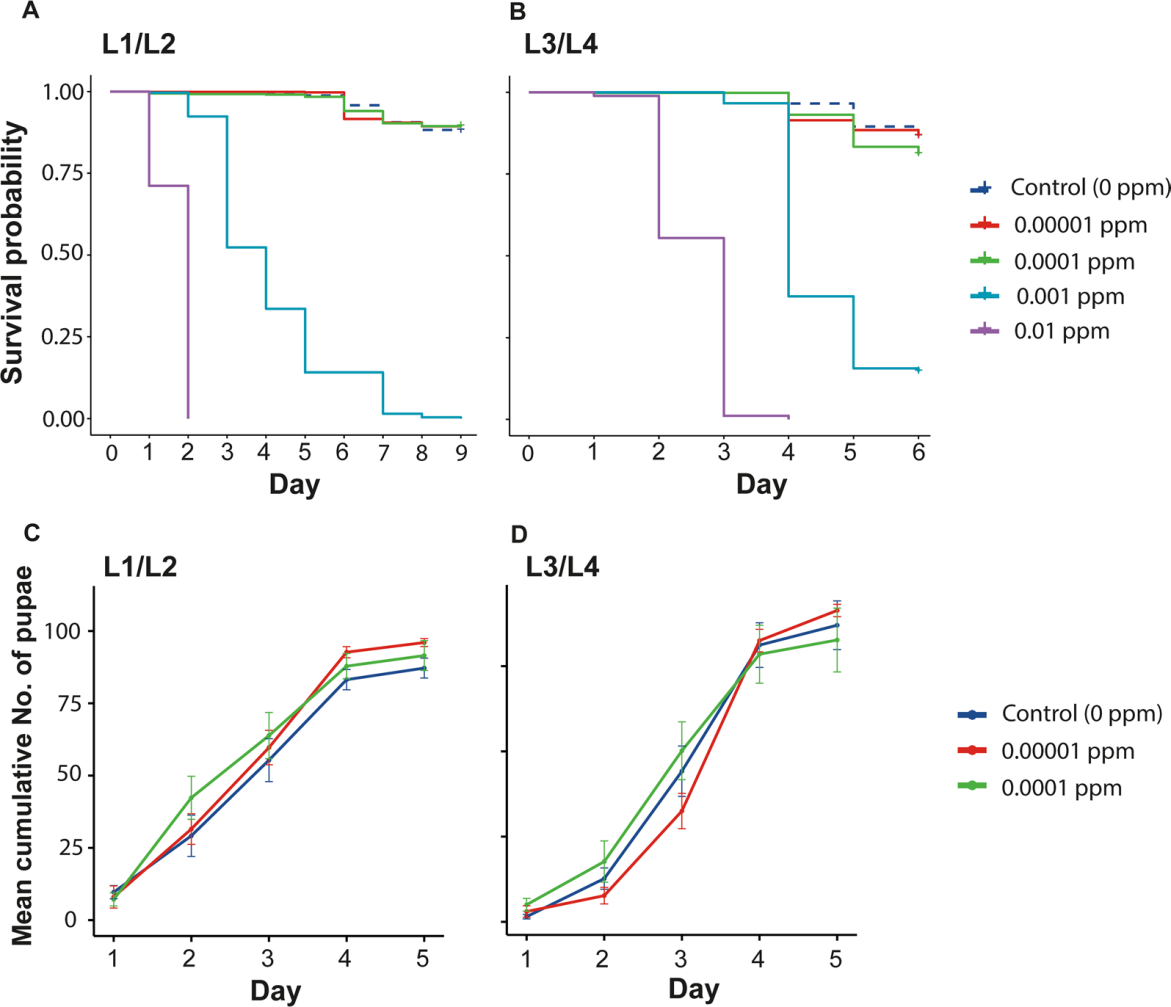
Survival and development of larvae in water containing different concentrations of ivermectin. **A** Survival of larvae exposed from the L1/L2 stage to ivermectin. **B** Survival of larvae exposed from the L3/L4 stage to ivermectin. **C** Pupation rate in larvae exposed from the L1/L2 stage to ivermectin. **D** Pupation rate in larvae exposed from the L3/L4 stage to ivermectin. For pupation rate, results of six rearing trays (2 biological replicates each with 3 technical replicates) are plotted as mean values ± SEM. *L1/L2* 1st/2nd instar larvae, L3/L4-3rd/4th instar larvae, *ppm* part per million

To determine the sublethal dose of ivermectin, larval survival and development were monitored after continued exposure to four concentrations of ivermectin: 0.01, 0.001, 0.0001 and 0.00001 ppm. Overall, independent of the stage at which larvae were exposed to ivermectin, a dose-dependent survival was observed with survival declining as the concentration of ivermectin increased (Fig. 1a, b). Larval survival probability was > 0.50 only for the 0.00001 and 0.0001 ppm concentrations, leading to their selection as the optimal sublethal doses (Table 1). To access larval development time, the pupation time was evaluated (Fig. 1c, d). Generalized estimating equation regression (GEE) model with Poisson as the family link was used to evaluate the relationship between ivermectin treatment and pupation time. For larvae exposed to ivermectin at the L1/L2 stage, pupation was faster in larvae exposed to 0.00001 ppm (coefficient = 4,67, 95% CI = 3.30–6.03, *p* ≤ 0.001) and 0.0001 ppm (coefficient = 5.63, 95% CI 4.25–7.01, *p* ≤ 0.001) ivermectin. For larvae exposed to ivermectin at the L3/L4 stage, pupation was slower in 0.00001(coefficient = − 1.90, 95% CI − 3.16–0.64, *p* ≤ 0.001) but faster in 0.0001 ppm (voefficient = 1.50,67, 95% CI 0.22–2.78, *p* ≤ 0.02).

**Table 1:** Survival probability and median survival time

Ivermectin concentration (ppm)	Survival probability (95% CI)	Median survival time	*p*-value
L1/L2			
0	0.880 (0.855–0.907)	–	–
0.00001	0.893 (0.869–0.917)	–	0.62
0.0001	0.893 (0.869–0.917)	–	0.62
0.001	0	4	< 0.001
0.01	0	2	< 0.001
L3/L4			
0	0.866 (0.839–0.893)	–	–
0.00001	0.866 (0.840–0.893)	–	0.8867
0.0001	0.810 (0.780–0.842)	–	0.0089
0.001	0.146 (0.111–0.192)	4	< 0.001
0.01	0	3	< 0.001

*L1/L2* 1st/2nd instar larvae, *L3/L4* 3rd/4th instar larvae, *ppm* part per million, *CI* confidence interval. *P*-value shows pairwise comparisons of larval survival probability between the control (no ivermectin treatment) and respective treated group

### Effects of exposure to sublethal concentration of ivermectin on fecundity and fertility

Following the identification of 0.00001 and 0.0001 ppm ivermectin as the sub-lethal concentrations, larvae were continually exposed to water with these concentrations, and the fecundity and fertility of the emerging mosquitoes were assessed. First, the time taken for blood-fed females to oviposit was evaluated. Relative to the control, exposure to sublethal concentrations of ivermectin in both the L1/L2 and L3/L4 stages reduced the time taken to oviposition by adult female mosquitos. Exposure to ivermectin at the L1/L2 reduced the mean time to oviposition of adult females from 4.53 (95% CI 4.08–4.98) days to 3.78 (95% CI 3.51–4.06) and 4.34 (95% CI 4.02–4.65) days in 0.00001 and 0.0001 ppm, respectively, though a significant reduction was only observed in the 0.00001 ppm (Table 2). Exposure to ivermectin at the L3/L4 stages reduced mean time taken to oviposition from 4.47 (95% CI 4.04–4.90) days to 4.11 (95% CI 3.78–4.44) and 3.86 (95% CI 3.60–4.12) days in 0.00001 and 0.0001 ppm, respectively with significant reduction only observed in 0.0001 ppm (Table 2).

**Table 2:** Time taken to oviposition post continued exposure to different concentrations of ivermectin

Ivermectin concentration (ppm)	*n*^a^	Biological rep^b^	Mean time to oviposition (95%CI)^c^	Coefficient (SE)	95% CI	*p*-value
L1/L2						
0 (Control)	70	2	4.53 (4.08–4.98)	–	–	–
0.00001	78	2	3.78 (3.51–4.06)	− 0.19 (0.07)	− 0.33–− 0.04	0.01
0.0001	86	2	4.34 (4.02–4.65)	− 0.05 (0.07)	− 0.18–0.08	0.48
L3/L4						
0 (Control)	62	2	4.47 (4.04–4.90)	–	–	–
0.00001	64	2	4.11 (3.78–4.44)	− 0.07 (0.09)	− 0.25–0.11	0.44
0.0001	72	2	3.86 (3.60–4.12)	− 0.14 (0.05)	− 0.24–− 0.03	0.01

Generalized negative binomial model with log link, dependent variable time (days), independent variable ivermectin concentration and biological replicate, clustered by technical replicate (*n* = 3)

*L1/L2* 1st/2nd instar larvae, *L3/L4-3rd/4th* instar larvae, *ppm* part per million

^a^Total number of mosquitoes followed up for time to oviposition (days), excluding mosquitoes that did not lay eggs

^b^Number of biological replicates overall per treatment group

^c^Adjusted incidence rate ratio and respective 95% confidence intervals

The proportion of mosquitoes that laid eggs following exposure to ivermectin at the larval stage ranged from 0.70–0.75 for those exposed at the L2/L2 and 0.60–0.74 for those exposed at the L3/L4 stage (Table 3). Relative to the control, exposure to ivermectin at the larval stages did not affect the proportion of mosquitoes that laid eggs (Table 3). Among the mosquitoes that laid eggs the number of eggs laid was counted for every female. No difference was observed in the average number of eggs laid per female mosquito (Table 4). Similarly, no difference was observed in the hatch rate per female (Table 5).

**Table 3:** Proportion of mosquitoes that laid eggs post continued exposure to different concentrations of ivermectin at the larval stages

Ivermectin concentration (ppm)	*N*^a^	Biological rep^b^	Number that laid eggs	Proportion that laid eggs (95% CI)	^c^OR (95%CI)	*p*-value
L1/L2						
0 (Control)	101	2	73	0.72 (0.63–0.80)	1	–
0.00001	112	2	78	0.70 (0.60–0.77)	0.68 (0.36–1.28)	0.23
0.0001	115	2	86	0.75 (0.66–0.82)	0.97 (0.51–1.82)	0.91
L3/L4						
0 (Control)	99	2	64	0.65 (0.55–0.73)	1	-
0.00001	106	2	64	0.60 (0.51–0.69)	0.81 (0.46–1.43)	0.47
0.0001	97	2	72	0.74 (0.65–0.82)	1.55 (0.84–2.88)	0.16

Binary logistic regression, dependent variable binary (oviposit or no eggs), independent variables ivermectin concentration (indicator) and biological replicate, clustered by technical replicate (*n* = 3). *L1/L2* 1st/2nd instar larvae, *L3/L4* 3rd/4th instar larvae, *ppm* part per million

^a^Number of emergent adult mosquitoes that blood fed after exposure to ivermectin during larval stages

^b^Number of biological replicates overall per treatment group

^c^Adjusted odds ratio

**Table 4:** Total eggs laid by individual adult mosquitoes post continued exposure to different concentrations of ivermectin at the larval stages

Ivermectin concentration (ppm)	Mean total eggs laid (95%CI)	Coefficient (SE)	95% CI	*p*-value
L1/L2				
0 (Control)	49.72 (42.04–57.40	Ref		
0.00001	47.27 (40.26–54.27)	− 2.45 (5.06)	− 12.40–7.50	0.63
0.0001	46.46 (40.28–52.64)	− 3.26 (5.03)	− 13.15–6.63	0.52
L3/L4				
0 (Control)	40.42 (33.23–47.62)	Ref		
0.00001	35.42 (28.88–41.95	− 5.01 (4.84)	− 14.54–4.52	0.30
0.0001	45.97 (39.31–52.63)	5.54 (4.95)	− 4.20–15.29	0.26

Generalized linear regression, dependent variable total eggs laid, independent variables ivermectin concentration (indicator) and biological replicate, clustered by technical replicate (*n* = 3). Total eggs laid (unhatched eggs plus hatched eggs)

*L1/L2* 1st/2nd instar larvae, *L3/L4* 3rd/4th instar larvae, *ppm* part per million, *SE* standard error, *CI* confidence interval

**Table 5:** Hatch rate of mosquitoes for individual adult mosquitoes post continued exposure to different concentrations of ivermectin at the larval stages

Ivermectin concentration (ppm)	Rep^a^	Proportion eggs hatched (95% CI)	^b^OR (95%CI)	*p*-value
L1/L2				
0 (Control)	2	0.36 (0.31–0.41)	Ref	–
0.00001	2	0.35 (0.32–0.39)	1.06 (0.81–1.39)	0.66
0.0001	2	0.30 (0.26–0.34)	0.79 (0.54–1.14)	0.21
L3/L4				
0 (Control)	2	0.34 (0.29–0.40)	Ref	–
0.00001	2	0.30 (0.25–0.35)	0.84 (0.60–1.17)	0.30
0.0001	2	0.35 (0.30–0.40)	1.06 (0.80–1.39)	0.69

Proportion of eggs hatched (hatched eggs/total eggs hatched) per individual mosquito. *L1/L2* 1st/2nd instar larvae, *L3/L4* 3rd/4th instar larvae, *ppm* part per million, *OR* odds ratio, *CI* confidence interval

^a^Number of biological replicates overall per treatment group

^b^Adjusted odds ratio

### Adult susceptibility to ivermectin after exposure to ivermectin at the larval stage

**Fig. 2:**
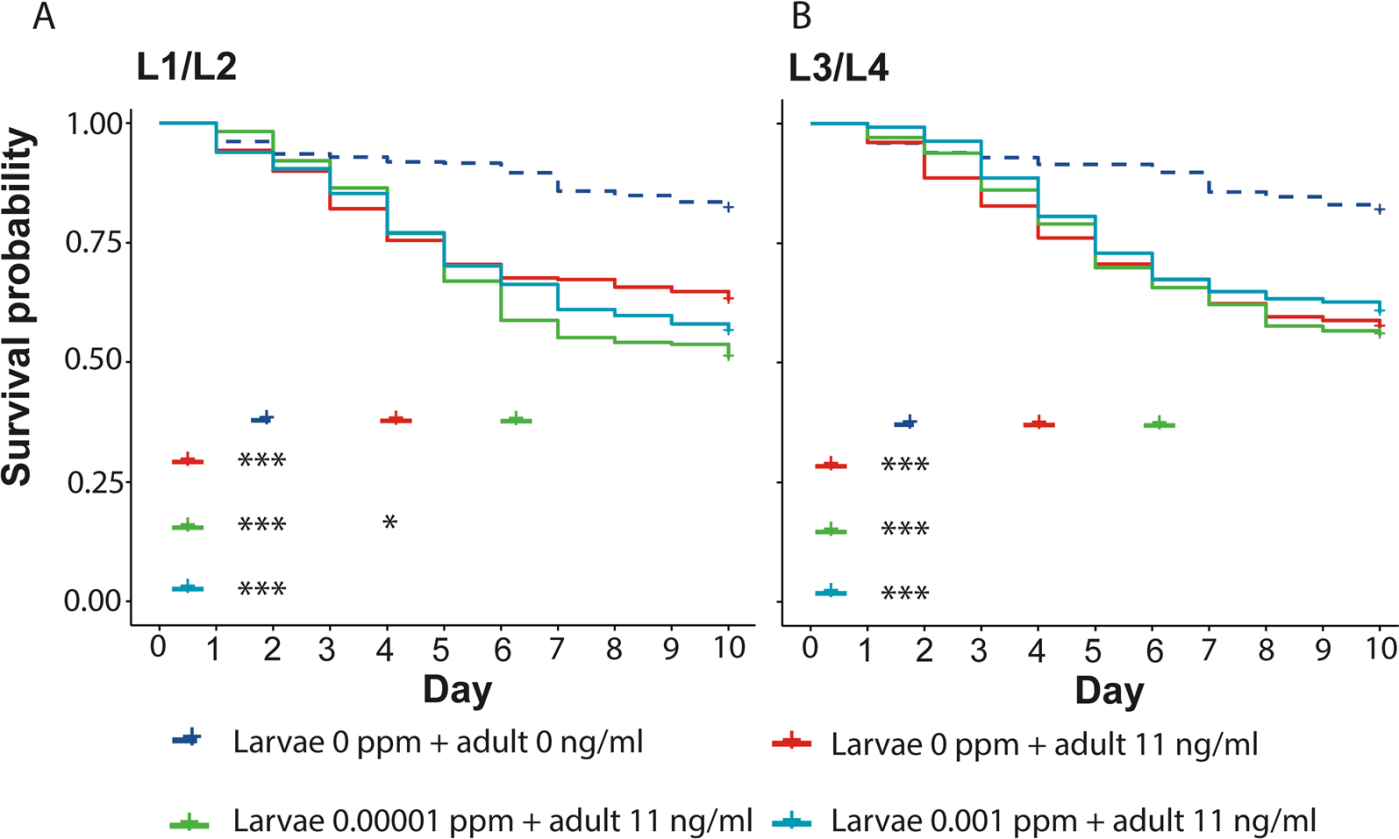
Adult susceptibility to ivermectin after exposure to ivermectin at the larval stage. The effect of larval ivermectin exposure at **A** L1/L2 and **B** L3/L4 stage on the adult’s susceptibility to ivermectin. Table inside the graph shows pairwise comparisons of survival for the different experimental conditions. Significance level indicated by asterisk; ***p < 0.01, *p < 0.05. *L1/L2* 1st/2nd instar larvae, *L1/L2* 3rd/4th instar larvae, *ppm* part per million

To determine whether exposure to ivermectin at the larval stage affected the susceptibility of the emerging adults, emerging adults were exposed to 11 ng/ml of ivermectin, a concentration that has previously been shown to yield at least 50% mortality in 10 days in this strain of mosquitoes [19]. Relative to the control, exposure to ivermectin at the L1/L2 larval stage yielded adult mosquitoes that were more susceptible to ivermectin. However, a significant susceptibility was only observed in the 0.00001 ppm concentration (log rank test: *p* = 0.024). Exposure to ivermectin at the L3/L4 stage did not affect the susceptibility of emerging adults to ivermectin (Fig. 2).

## Discussion

Ivermectin is one of the drugs under consideration by WHO for use as a systemic insecticide for the control of malaria [20]. This arises from the ability of ivermectin to kill adult malaria vectors that blood feed on a treated subject [4, 5]. In addition to mortality, ivermectin affects other biological functions in adults causing reduced fecundity and fertility further negatively impacting the capacity of *Anopheles* mosquitoes to transmit malaria [14, 15]. The implementation of ivermectin MDA for malaria control has been shown to affect the density of malaria vectors [21]. Though the reduction in vector density is attributed to the effects of ivermectin on the adult mosquitoes, it is important to understand whether the effect on larvae could indirectly contribute to the changes observed in adult mosquitoes, thus contributing to the overall effect on malaria transmission.

The amount of ivermectin that ends up in the larval habitats varies depending on ivermectin dosage administered, application form, environmental conditions as well as source of contamination [22]. The predicted environmental concentrations (PEC) for surface water contaminated with ivermectin through soil is estimated to range between 1e–7 to 0.0000072 ppm, which is much lower compared to contamination through direct excretion, which is estimated to range between 0.000209 to 0.000529 ppm [22]. In addition to being exposed to ivermectin-contaminated surface water, some malaria vectors could get exposed to higher levels of ivermectin as they have been reported to breed in close proximity to livestock, for instance in cattle hoof prints [23]. In the current study, a range of concentrations ranging from 0.01 ppm to 0.00001 ppm was tested. The highest concentration was selected based on a previous report of > 80% survival of *An. gambiae* 2 days post-exposure [13]. Similarly, Derua et al. (2016) reported > 80% survival in 3rd/4th instar larval stage 2 days post exposure [13]. However, by 4 days post exposure, larval mortality was 100%. In this this current study, exposure to the same concentration at the first/second instar larval stage yielded 100% mortality 2 days post exposure, suggesting greater susceptibility in our laboratory-reared strain and leading to selection of 0.0001 and 0.00001 ppm as the optimal sub-lethal doses since larvae survived and developed to the adult stage.

While *An. gambiae* larvae showed < 80% survival in concentration of > 0.001 ppm, in a previous study [19], adults from the same strain needed higher concentrations of ivermectin to achieve the same mortality, confirming that larval stages of *An. gambiae* are more susceptible to ivermectin compared to the adults. In Drosophila, this increased susceptibility in the larval stage is attributed to lower detoxification due to lower P-glycoprotein levels [24]. Whether the same physiological explanation applies to the differences in susceptibility between the *An. gambiae* adult and larval stages remains to be investigated. The differences in susceptibility to ivermectin does not only exist between the mosquito stages but also among the mosquito species with larvae of *Culex quinquefasciatus* being more susceptible than *An. gambiae* [13]. Previously, this had been hypothesized to be due to differences in ivermectin bioavailability for the two species with ivermectin being more available for culicines since they are bottom feeders [13]. However, it is possible that physiological differences between the two species relating to ivermectin absorption, metabolism and/or detoxification could be playing a role.

The present study revealed that exposure to ivermectin at the larval stages did not affect the number of eggs laid and hatch rate. This is contrary to what has been previously reported for *Cx. quinquefasciatus* [25]. In *Cx. quinquefasciatus*, exposure to ivermectin at the larval stages has been shown to affect egg development with the effect being associated with reduced cytoplasmic inclusions in the larval fat body cells, consequently affecting the egg mass and number of eggs in the adults [25]. Despite *An. gambiae* larvae being more susceptible to ivermectin than *An. gambiae* adults, exposure of adults to the same concentrations of ivermectin used in the current study also showed no differences in the number of eggs or hatchability of the eggs [4]. However, when exposed to higher concentrations, egg production was completely blocked [4]. These results suggest a threshold after which egg production is completely blocked, and any concentration below the threshold has no effect on the number and viability of the eggs produced. These findings have implications for the planning of potential ivermectin resistance management [26].

Exposure to ivermectin at the adult stages has been shown to delay defecation and re-feeding [27]. This could possibly be caused by delayed blood meal digestion consequently delaying egg production. Inversely, the current study reported a reduction in time taken to oviposition when larvae were exposed to ivermectin. Why exposure to ivermectin at the larval stage leads to a reduction in the time taken to oviposition remains puzzling and warrants further investigation as shortening the gonotrophic cycle would potentially affect the blood-feeding frequency of the adult mosquitoes and therefore affect vector human contact [28].

Exposure to insecticides in the larval stages has been reported to increase tolerance of the adult mosquitoes to insecticides [29, 30]. In the current study, exposure to ivermectin in the third/fourth-instar larvae did not affect the susceptibility of the adults to ivermectin. However, exposure at the first/second-instar larvae increased susceptibility to ivermectin in the adults though this effect was only observed when larvae were exposed to the low concentration of ivermectin.

## Conclusion

In summary, this study shows that contamination of larval habitats with ivermectin affects *An. gambiae* larval survival and potentially has an impact on public health. The number of eggs and viability of the eggs in the adults emerging from ivermectin contaminated habitats remain unaffected. Lastly, exposure to ivermectin at the larval stages does not compromise the efficacy of ivermectin in the emerging adults. However, these results are based on a short exposure period and within a controlled environment. In nature, prolonged ivermectin use (repeated MDAs) could result in bioaccumulation of drug residues and selection of traits that may be consequential for malaria vector control. Therefore, further studies examining the impacts of long-term exposure of malaria vectors to sub-lethal doses of ivermectin are warranted.

## Data Availability

All data are included in this published article.

## Abbreviations

L1/L2: 1st/2nd instar larvae
L3/L4: 3rd/4th instar larvae
ppm: Part per million
ng/ml: Nanogram/milliliter
